# Editorial: Insights into bee diseases and bee health

**DOI:** 10.3389/fcimb.2022.993440

**Published:** 2022-08-05

**Authors:** Giovanni Cilia, Mario Forzan

**Affiliations:** ^1^ CREA Research Centre for Agriculture and Environment, Bologna, Italy; ^2^ Department of Veterinary Sciences, University of Pisa, Pisa, Italy

**Keywords:** bee health, biodiversity, spillover, pathogen transmission, one-health approach

The decline of bees is an important worldwide diffused event, causing severe ecological damage (O’Neal et al., 2018; Siviter et al., 2018; Hrynko et al., 2021). The causes of this decline are strictly related to habitat loss, climatic change, pesticides, parasites, and diseases (Duchenne et al., 2020; Hofmann and Renner, 2020; Wenzel et al., 2020; Cane, 2021; Hung et al., 2021; Librán-Embid et al., 2021). The spread of pathogens alone plays a pivotal role to endanger pollinators. Besides, the spillover of pathogens from honey bees (*Apis mellifera* L.) to other bee species increases this decline (Nanetti et al., 2021). The circulation of pathogens within bees can happen by different routes: horizontally (via flowers, pollen, predation or direct contact), vertically or sexually (Chen et al., 2006; Mazzei et al., 2014; Forzan et al., 2017; Gruber et al., 2017; Mazzei et al., 2018; Mazzei et al., 2019; Schittny et al., 2020). Some aspects of the dynamics of host-pathogen interaction in honey bees are still unknown and therefore it is very important to clarify them to adopt possible prevention measures.

Toward the end, the Research Topic has brought together the results of five studies by 32 authors from six different countries (Egypt, India, Italy, Saudi Arabia, Spain, and United States) demonstrating not only the breadth of the field but also its international relevance. Areas covered include the effect of *Nosema ceranae* infection related to the age of honey bees and season (Jabal-Uriel et al.), the gut microbiome of *Apis florea* (Ganeshprasad et al.), the microRNA profile of the mite *Varroa destructor* (Kumar et al.), the presence of honey bee virus in *Vespa orientalis* (Power et al.) and the spillover of honey bee pathogens in wild pollinators (Cilia et al.). All 5 published articles explored this theme and emphasize the importance of this Research Topic (**Figure 1**).

**Figure 1:**
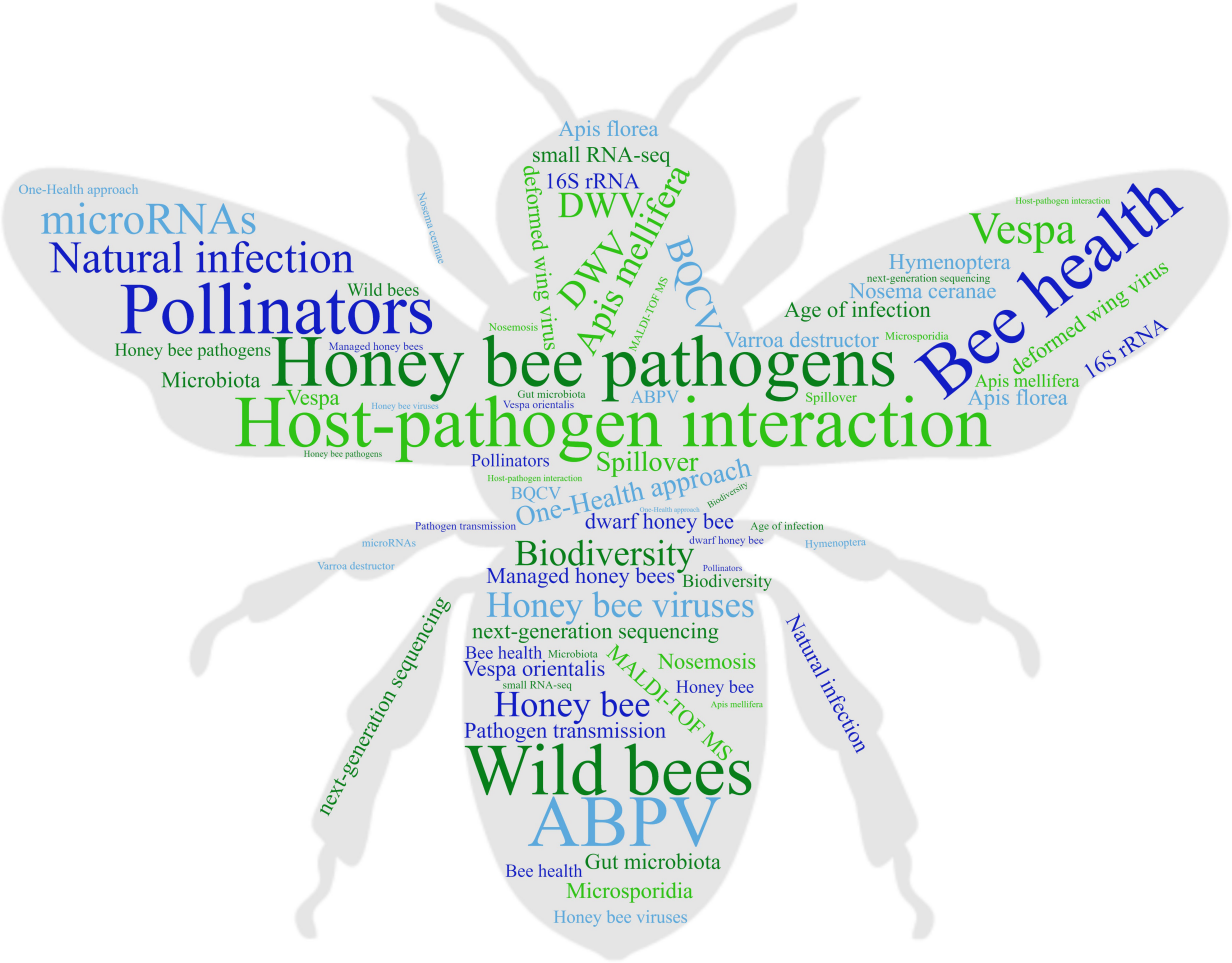
A word cloud created from the titles and keywords of every article published on the Research Topic.

The relationship between the honey bees’ age, the season and the onset of *Nosema ceranae* infection was investigated by Jabal-Uriel et al. Using marked adult honey bee workers less than 24h of age introduced into six different recipient colonies naturally infected with *N. ceranae* in spring and autumn, the spread of nosemosis was clarified. Workers of known age were individually analyzed to evaluate the infection by the microsporidia. In spring and autumn, honey bees were naturally infected by *N. ceranae* after 5 and 4 days post-emergence, respectively. Also, in-hive prevalence increased from that point onwards, reaching the highest mean infection on day 18 post-infection in spring. The probability of infection increased significantly with age in both seasons even if the age variable was better correlated in spring. The *N. ceranae* load increased with age in both seasons although the relationship between age and load was clearer in spring than in autumn. The study has revealed that both age and season play an important role in the probability and the development of *N. ceranae* infection in honey bee colonies, bringing important information to understand how the infection spreads inside the hive

Ganeshprasad et al. investigated for the first time the gut microbiome of *Apis florea*, the most primitive wild honey bee indigenous to the Indian subcontinent. The study reported the analysis and the identification of gut bacteria in *A. florea* individuals, employing culture-based and culture-independent methods, identifying the bacterial strains using MALDI-TOF MS and 16S rRNA sequencing. This study has split gut bacteria into four bacterial phyla, four families, and 10 genera. The dominant taxa identified in *A. florea* belonged to the family Enterobacteriaceae (79.47%), followed by Lactobacillaceae (12.75%), Oxalobacteraceae (7.45%), and Nocardiaceae (0.13%). The prevailing bacteria belonged to *Enterobacter, Lactobacillus, Escherichia-Shigella, Massilia, Klebsiella, Citrobacter, Pantoea, Serratia, Rhodococcus*, and *Morganella* genera, belonging to phyla Proteobacteria, Firmicutes, Actinobacteria, and Bacteroidetes. This study observed the occurrence of a few bacteria that were not previously reported for their occurrence in other species of the Apis genus, demonstrating the importance of this investigation in the bee microbiome.

The exotic mite *Varroa destructor* is one of the most known honey bee ectoparasites and vector of Deformed Wing Virus (DWV). The *Varroa*-DWV complex is one of the most important causes of the annual collapse of the honey bees’ colonies. MicroRNAs (miRNAs) are made up of 22-24 nucleotides non-coding RNAs produced by all plants and animals and some viruses influencing biological processes through post-transcriptional regulation of gene expression. Knowledge of miRNAs and their function in mite biology remains limited. In the study reported by Kumar et al., small RNA libraries from male and female *V. destructor* were constructed using Illumina’s small RNA-Seq platform. A total of 101,913,208 and 91,904,732 small RNA reads (>18 nucleotides) from male and female mites were analyzed using the miRDeep2 algorithm. A conservative approach predicted 306 miRNAs, 18 of which were upregulated and 13 downregulated in *V. destructor* females compared with males, whose expressions were validated by quantitative real-time PCR. This dataset provides a list of potential miRNA targets involved in regulating vital *Varroa* biological processes and paves the way for developing strategies to target Varroa and their viruses (Kumar et al.).

Power et al. investigated the possible role of *Vespa orientalis* as a vector for honey bee viruses. The Oriental hornet (*V. orientalis*) is spreading across the Italian territory threatening the health and wellbeing of honey bee colonies. Although no macroscopical alterations were found in the 30 collected adult individual biomolecular results showed that DWV was the most detected virus (83.3%). Several viral co-infections were found in 20 samples (66.7%). The most frequent (56.7%) was the association between DWV and Acute bee paralysis virus (ABPV), often associated with black queen cell virus (BQCV) (30.0%). One sample (3.33%) showed the co-infections of DWV, ABPV, BQCV and Kashmir bee virus (KBV). The detected viruses are the most widespread in the Italian apiaries, suggesting the possible transmission from honey bees to *V. orientalis*, probably predating infected adult workers or cannibalizing their carcasses. However, to date, even if it is still not clear if those viruses are replicative in *V. orientalis*, they can still act as a mechanical vector in the environment.

The possible spillover of pathogens from honey bees to wild pollinators was investigated in the North of Italy. The prevalence and abundance of 21 honey bee pathogens (11 viruses, 4 bacteria, 3 fungi, and 3 trypanosomatids) were assessed in the flower-visiting entomofauna sampled from March to September 2021 in seven sites in two North-Italian regions (Emilia-Romagna and Piedmont). A total of 1,028 specimens were collected, identified, and analysed. Out of the twenty-one pathogens that were searched for, only thirteen were detected. The prevalence of the positive individuals reached 63.9%, for *N. ceranae*, DWV, and CBPV as the most prevalent pathogens. In general, the pathogen abundance averaged 5.15 * 10^6^ copies, with CBPV, *N. ceranae*, and BQCV as the most abundant pathogens, with 8.63, 1.58, and 0.48 * 10^7^ copies, respectively. All the detected viruses were found to be replicative. The sequence analysis indicated that the same genetic variant was circulating in a specific site or region, suggesting that interspecific transmission events among honey bees and wild pollinators could be possible. Frequently, *N. ceranae* and DWV were found co-infecting the same individual. The circulation of honey bee pathogens in wild pollinators was never investigated before in Italy. For the first time, this study highlighted honey bee pathogens infection in 72 new wild pollinator species (Cilia et al.).

All of the published articles encourage the implementation of studies on bee health and the host-pathogen interactions to increase our understanding of their environmental implications, embracing a One Health approach to pollinators’ welfare (Wilfert et al., 2021).

## Author contributions

Both authors listed have made a substantial, direct, and intellectual contribution to the work and approved it for publication.

## Acknowledgments

We would like to thank all the authors who contributed their papers to this Research Topic, the editors and the reviewers for their helpful recommendations.

## Conflict of interest

The authors declare that the research was conducted in the absence of any commercial or financial relationships that could be construed as a potential conflict of interest.

## Publisher’s note

All claims expressed in this article are solely those of the authors and do not necessarily represent those of their affiliated organizations, or those of the publisher, the editors and the reviewers. Any product that may be evaluated in this article, or claim that may be made by its manufacturer, is not guaranteed or endorsed by the publisher.
